# Correction: WHO target product profile for TB detection at peripheral settings: 2024 update

**DOI:** 10.1371/journal.pgph.0005315

**Published:** 2025-10-09

**Authors:** Mikashmi Kohli, Alexei Korobitsyn, Nazir Ismail, Matteo Zignol, Tereza Kasaeva, Puneet Dewan, Morten Ruhwald, the Scientific TPP Development group

The following information is missing from the Acknowledgement statement. The statement should begin with this sentence: We would like to especially thank Alexandra de Nooy, Brooke Nichols, and Tom Ockhuisen (University of Amsterdam, Netherlands) for conducting the modeling work that informed this update. This work has been published in detail elsewhere [8].

In the Fig 2 caption, there is a missing citation. Please see the complete, correct Fig 2 caption here.

In the Cost subsection of the Results, the third sentence of the fourth paragraph should have cited reference 9 instead of 8.

The correct sentence should read: Given that we would expect a greater number of tests to be done, the cost per test would need to decrease to achieve cost neutralization. Details of this work have been published elsewhere [9].

**Fig 2:**
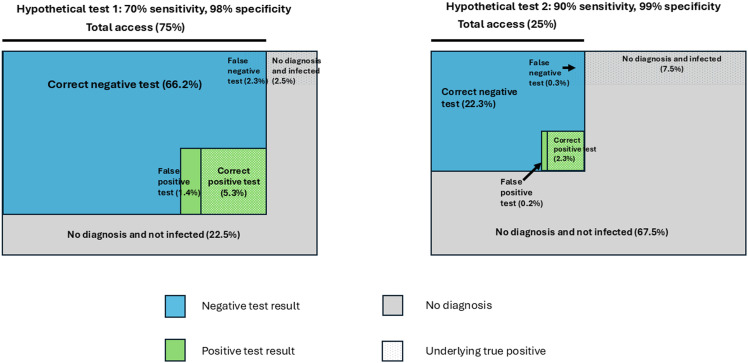
Trade-off between test accuracy and test access, assuming an underlying disease prevalence of 10%. Adapted from De Nooy et al [8].
