# Supplementary material for: WHO target product profile for TB detection at peripheral settings: 2024 update
Source: PLOS Glob Public Health. 2025 Jun 11;5(6):e0004612. doi: 10.1371/journal.pgph.0004612 (PMC12157113; doi:10.1371/journal.pgph.0004612)
Supplement: Attachment — Submitted filename: Response to the reviewers.docx [file pgph.0004612.s003.docx]

| **Reviewer 1** |  |
| --- | --- |
| Clear and accurate description of the process to develop WHO target product profiles. A reference to the WHO TPP Directory could have been included: <https://www.who.int/our-work/science-division/research-for-health/target-product-profile-directory> and I'd ensure that the original TPP is uploaded in the Directory as it is an additional way to disseminate this product to relevant audience members. | We thank the reviewer for their comments, careful review and for updating this useful directory.  This directory has been cited in the text and the references (ref:7). |
| **Reviewer 2** |  |
| 1. Figure 4b) is not clear. Their should be a brief explanation with the figure. | We have added text to the legend of the Figure 4b. It now says, “This graph shows geographical representation of the respondents from the public comment to the TPP draft  The intensity of purple shading in the graph represents the number of respondents from each country. “ |
| 2. There are limited respondents from Africa and South East Asia. Would the data provided accurately represent these regions, especially in terms of pricing of tests ? | We agree that there are fewer respondents from these regions, however, we did have good representation from south Africa, India, Indonesia, Nigeria, Ethiopia and other countries as well.  However, the data in the TPP document is not just from public comments. The TPP scientific committee has very good representation across the globe and that helps us give a generalizable document which is acceptable to not just experts in this field but also representatives from civil society, advocacy groups, policymakers etc.  However, we understand that there should be more outreach at the public comment stage to ensure we have a better representation at that stage of TPP process and there is work ongoing for this at the WHO |
| 3. It would be good to mention the various tests considered in sputum and non-sputum POC, near POC and low complexity assays. | During the TPP development process, we do not refer to any specific tests as it is more forward-looking, test agnostic and not favoring any specific company or test type. Rather the idea of this TPP update was to work on where we place the test and specimen type to allow access to these tests. While updating this TPP, the committee kept in mind the products in the pipeline and also making it aspirational for innovators.  However, during the discussion, there were many existing tests that were mentioned such as the existing WHO recommended molecular tests, upcoming tests on swabs for molecular tests and new LAM tests. |
| **Editor/ Journal Requirements** |  |
| 1. Please provide additional details regarding participant consent. In the ethics statement in the Methods and online submission information, please ensure that you have specified (1) whether consent was informed and (2) what type you obtained (for instance, written or verbal, and if verbal, how it was documented and witnessed). If your study included minors, state whether you obtained consent from parents or guardians. If the need for consent was waived by the ethics committee, please include this information.    If you are reporting a retrospective study of medical records or archived samples, please ensure that you have discussed whether all data were fully anonymized before you accessed them and/or whether the IRB or ethics committee waived the requirement for informed consent. If patients provided informed written consent to have data from their medical records used in research, please include this information. | We provide the following text under ethical considerations, “All members of the STDG made a declaration on potential interests, which were reviewed and managed by WHO; a statement on the declarations is available in the WHO TPP document^9^. As member of the WHO TPP Development group, and its core task being to provide technical expertise for the TPP document, ethics approval was not needed. These tasks were included in the declaration forms managed by the WHO to obtain informed consent in a written format from the TPP development group. Additionally, during the public comment process, information on this process was shared on the WHO webpage and as a standard practice. This also included getting an approval from the participants to share their comments and personal information for analysis. If the participants did not approve of sharing their responses and personal information, they were not included in the analyses.”.  It was informed consent in a written format. |
| 2. Please provide separate figure files in .tif or .eps format.    For more information about figure files please see our guidelines:  LINK  https://journals.plos.org/globalpublichealth/s/figures  https://journals.plos.org/globalpublichealth/s/figures#loc-file-requirements | We have uploaded these images in the required format and dpi requirements. |
| 3. Please upload a copy of Figures 1, 2, 3, 4a and 4b which you refer to in your text on page 5,6, 7, 8. Or, if the figure is no longer to be included as part of the submission please remove all reference to it within the text. | We have uploaded these images. |
| 4. In the online submission form, you indicated that All data has been provided as part of this manuscript and any additional information can be provided on request as well.    All PLOS journals now require all data underlying the findings described in their manuscript to be freely available to other researchers, either  1. In a public repository,  2. Within the manuscript itself, or  3. Uploaded as supplementary information.    This policy applies to all data except where public deposition would breach compliance with the protocol approved by your research ethics board. If your data cannot be made publicly available for ethical or legal reasons (e.g., public availability would compromise patient privacy), please explain your reasons by return email and your exemption request will be escalated to the editor for approval. Your exemption request will be handled independently and will not hold up the peer review process, but will need to be resolved should your manuscript be accepted for publication. One of the Editorial team will then be in touch if there are any issues. | All information has been provided in the manuscript and supplementary data. |
| 5. Figure 4b: please (a) provide a direct link to the base layer of the map (i.e., the country or region border shape) and ensure this is also included in the figure legend; and (b) provide a link to the terms of use / license information for the base layer image or shapefile. We cannot publish proprietary or copyrighted maps (e.g. Google Maps, Mapquest) and the terms of use for your map base layer must be compatible with our CC-BY 4.0 license.    Note: if you created the map in a software program like R or ArcGIS, please locate and indicate the source of the basemap shapefile onto which data has been plotted.    If your map was obtained from a copyrighted source please amend the figure so that the base map used is from an openly available source. Alternatively, please provide explicit written permission from the copyright holder granting you the right to publish the material under our CC-BY 4.0 license.    Please note that the following CC BY licenses are compatible with PLOS license: CC BY 4.0, CC BY 2.0 and CC BY 3.0, meanwhile such licenses as CC BY-ND 3.0 and others are not compatible due to additional restrictions.    If you are unsure whether you can use a map or not, please do reach out and we will be able to help you. The following websites are good examples of where you can source open access or public domain maps:  * U.S. Geological Survey (USGS) - All maps are in the public domain. (http://www.usgs.gov)  * PlaniGlobe - All maps are published under a Creative Commons license so please cite “PlaniGlobe, http://www.planiglobe.com, CC BY 2.0” in the image credit after the caption. (http://www.planiglobe.com/?lang=enl)  * Natural Earth - All maps are public domain. (<http://www.naturalearthdata.com/about/terms-of-use/>) | For Figure 4b, the base map was developed using the Microsoft Excel Maps function.  We have also mentioned it in the footnote now as the source. |
| 6. Please review your reference list to ensure that it is complete and correct. If you have cited papers that have been retracted, please include the rationale for doing so in the manuscript text, or remove these references and replace them with relevant current references. Any changes to the reference list should be mentioned in the rebuttal letter that accompanies your revised manuscript. If you need to cite a retracted article, indicate the article’s retracted status in the References list and also include a citation and full reference for the retraction notice. | All references are correct and cited. We have also added one more reference as per the reviewer and updated the numbers. |
